# *Crassostrea gigas* mortality in France: the usual suspect, a herpes virus, may not be the killer in this polymicrobial opportunistic disease

**DOI:** 10.3389/fmicb.2015.00686

**Published:** 2015-07-06

**Authors:** Bruno Petton, Maxime Bruto, Adèle James, Yannick Labreuche, Marianne Alunno-Bruscia, Frédérique Le Roux

**Affiliations:** ^1^LEMAR UMR 6539, IfremerArgenton-en-Landunvez, France; ^2^Unité Physiologie Fonctionnelle des Organismes Marins, IfremerPlouzané, France; ^3^CNRS, Equipe Génomique des Vibrios, LBI2M, UPMC Paris 06, UMR 8227, Integrative Biology of Marine Models, Sorbonne UniversitésRoscoff, France

**Keywords:** pacific oysters, summer mortality, herpes virus, vibrio pathogenicity, experimental infection

## Abstract

Successive disease outbreaks in oyster (*Crassostrea gigas*) beds in France have resulted in dramatic losses in production, and subsequent decline in the oyster-farming industry. Deaths of juvenile oysters have been associated with the presence of a herpes virus (OsHV-1 μvar) and bacterial populations of the genus *Vibrio*. Although the pathogenicity of OsHV-1 μvar, as well as several strains of *Vibrio* has been demonstrated by experimental infections, our understanding of the complexity of infections occurring in the natural environment remains limited. In the present study, we use specific-pathogen-free (SPF) oysters infected in an estuarine environment to study the diversity and dynamics of cultured microbial populations during disease expression. We observe that rapid *Vibrio* colonization followed by viral replication precedes oyster death. No correlation was found between the vibrio concentration and viral load in co-infected animals. We show that the quantity of viral DNA is a predictor of mortality, however, in the absence of bacteria, a high load of herpes virus is not sufficient to induce the full expression of the disease. In addition, we demonstrate that juvenile mortalities can occur in the absence of herpes virus, indicating that the herpes virus appears neither essential nor sufficient to cause juvenile deaths; whereas bacteria are necessary for the disease. Finally, we demonstrate that oysters are a reservoir of putative pathogens, and that the geographic origin, age, and cultivation method of oysters influence disease expression.

## Introduction

The oyster aquaculture industry in France has been shaped by various infectious diseases caused by bacteria, viruses, or parasites (Grizel and Héral, 1991; Cochennec et al., 2000; Le Roux et al., 2001). Today, cultivation of the imported species, *Crassostrea gigas* (Pacific oyster), constitutes the vast majority of oyster farming (Rohfritsch et al., 2013), and diseases affecting this species have been steadily rising over the past decades, threatening the long-term survival of farmed and natural stocks. For example, a syndrome known as “summer mortality” has been affecting oyster production on the west coast of France (Samain, 2008). This syndrome has been associated with an ostreid herpes virus, designated OsHV-1, and bacteria of the genus *Vibrio* as infectious agents (Samain, 2008). “Summer mortality” is also thought to be influenced by elevated temperature (>19°C), physiological stress associated with maturation, genetic traits of the host, and aquaculture practices. However, none of these individual factors have been shown to consistently be responsible for the syndrome.

In addition to “summer mortality,” since 2008, juvenile oyster (<12 months) deaths have increased considerably, and current mortality levels range between 60 and 90% (Martenot et al., 2011). These events are more pervasive because they occur at a lower threshold temperature (16°C) and geographically extend to all coastal regions in France (Atlantic, Channel, and Mediterranean; Pernet et al., 2012; Petton et al., 2013). This dramatic mortality level coincides with an increase in the detection frequency of herpes virus and with the identification of OsHV-1 variants, the OsHV-1 μvar being the most predominant genotype (Segarra et al., 2010; Martenot et al., 2011, 2013). It has been postulated that the variant OsHV-1 μvar is more virulent than the reference strain (OsHV-1) and could be the etiological agent of juvenile mortalities (Segarra et al., 2010; Martenot et al., 2011, 2013; Schikorski et al., 2011a,b). However, these assumptions are speculative (to the authors’ knowledge, no published data has formally demonstrated that the pathogenicity of OsHV-1 μvar is higher than that of OsHV-1) and await further confirmations. In particular, recent technological improvments in the procedures used to detect the viral DNA load in animal tissues now offer an opportunity revisiting these assumptions. The real-time PCR-based assays recently developed and now widely used as detection tools have been shown to be at least two orders of magnitude more sensitive than earlier quantitative PCR assays (Arzul et al., 2002; Pepin et al., 2008; Martenot et al., 2010). Hence, the determination of the viral DNA quantity in archived samples (collected before 2008) using the older and newer protocols should be performed to verify that the increase of mortality correlates with an increase in virus prevalence. Therefore, it is yet to be determined if the increase in juvenile oyster mortalities since 2008 corresponds to a new disease or to a worsening summer mortality syndrome, emphasizing the need to continue investigating the diversity and functioning of all oyster disease agents.

This is particularly important for oyster farming because the only regulation implemented to help the oyster industry to date is based on the hypothesis that juvenile mortalities are due to the emergence of a new disease connected to OsHV-1 μvar. The European Union Regulation (175/2010), implemented in March of 2010, suggests that when the presence of OsHV-1 μvar is detected, disease control measures should be implemented, including the establishment of a containment area to restrict the movement of *C. gigas* oysters. Thus, demonstrating that the herpes virus is a marker of the disease and/or the unique etiological agent requires further investigations.

Previous research efforts have mainly focused on the viral hypothesis and knowledge on the role of bacteria remains limited. We recently investigated the oyster disease ecology of microdiverse *Vibrio* genotypes using a new field-based approach (Lemire et al., 2014). We took advantage of recently developed specific-pathogen-free (SPF) juveniles of *C. gigas*, which become naturally infected when placed in an estuarine environment (Petton et al., 2013). We showed that the onset of disease in oysters is associated with progressive replacement of diverse and benign colonizers by members of a non-clonal but phylogenetically coherent virulent population of *Vibrio crassostreae*. While the detection of putative pathogens (OsHV-1-μvar and *V. crassostreae*) coincides with oyster deaths, the respective role of each infectious agent in the disease remains unresolved. When oysters are co-infected by the herpes virus and vibrios, one can ask if a synergy between these microbes occurs and, if so, is required to the disease. Finally as the European Union regulation implies the establishment of containment areas of infected oysters their role as reservoirs of pathogens needs to be demonstrated.

In the present study, we investigated the respective roles of herpes virus and vibrios in causing oyster mortality. The first issue we address is the potential for co-infection by the herpes virus and *Vibrio* spp. in oysters infected in the field. To this end, we use standardized SPF oysters, descendants of a pool of genitors produced in hatchery under highly controlled conditions, in order to decrease the influence of genetic and environmental parameters that could affect the host sensitivity to the disease. SPF oysters represent a suitable model to investigate the role of each pathogenic agent in the disease process, because the vibrio fraction is low in SPF oyster tissue and herpes virus undetected. Second, we investigate whether the quantity of virus and/or *Vibrio* spp. in infected oysters can be used as a predictor of mortality. Third, we aim at differentiating the roles of *Vibrio* spp. and the herpes virus as causative agents of the disease. Finally, we investigate the role of oysters as a reservoir of putative pathogens.

## Materials and Methods

### Specific-Pathogen-Free Juveniles

Wild oyster spats were collected in Fouras (Marennes-Oléron, France) in 2011 and were moved to grow-up areas located at Paimpol and at Aber Benoît (northern Brittany, France) between 2012 until 2014. These animals were exposed to diseases during the spring 2012 and suffered mortality >50% (Petton et al., 2013). In January and in April 2014, 60 individuals were successively transferred to the experimental Ifremer facilities located at Argenton (Brittany, France) and treated by chloramphenicol (8 mg/L) for 5 days prior to maturation conditioning as described previously (Petton et al., 2013). After gamete stripping and fertilization, animals were reared under controlled conditions up to 5 months, or until reaching a mean individual wet mass between 0.5 and 3.0 g. Quantification of the herpes virus was performed by qPCR at the different steps of the SPF production, i.e., from the broodstock at the beginning of their conditioning to the larval and post-larval stages and spats.

### Natural Infection in the Field and Cohabitation Experiments in the Laboratory

SPF oysters were maintained in Bay of Brest (Pointe du Château, 48° 20′ 06.19″ N, 4° 19′ 06.37″ W) during a disease outbreak (seawater temperature > 16°C). Total mortalities resulting in the field were recorded daily for 57 days (from 7 August 2014 to 3 October 2014). For cohabitation experiments, field-exposed oysters (i.e., “donors”) were brought back to the laboratory and cohabited in a tank with SPF oysters (i.e., “recipients”) at 21°C under controlled conditions of water renewal (Petton et al., 2015). When indicated, chloramphenicol (8 mg/L) was added in tanks every 2 days to remove the cultivable microbiota in the oyster tissues. Alive and dead donors and recipients were daily counted, and dead animals were removed from the tanks.

### Bacterial Isolation and Identification

Oysters were sacrificed, dissected to remove the digestive gland, and ground in sterile seawater (10 mL/g of wet tissue). The total cultivable bacteria and *Vibrio* microbiota was quantified (cfu/mg of tissue) using serial dilutions on Marine agar (Difco) and TCBS selective media, respectively. In addition, in two distinct experiments, the hemolymph of 40 field-exposed oysters (8 to 11 July; 25 to 29 July 2014) was non-destructively collected from marked individuals that were held for 10 days in 1-L tanks to identify animals that died or survived. Vibrios isolated from the hemolymph were quantified on TCBS. Randomly selected colonies (∼80/from 4 animals that died after 24 h; ∼80/from 4 animals that survive after 9 days) were re-streaked two times on TCBS, cultivated in Zobell media (4 g/L bactopeptone and 1 g/L yeast extract in artificial sea water, pH 7.6) and stored at -80°C. For DNA sequencing, purified isolates were grown in Zobell overnight and their DNA was extracted using a DNA extraction kit (Wizard, Promega). The partial *hsp60* gene was amplified for all isolates as described previously (Hunt et al., 2008). The PCR conditions were: 3 min at 95°C followed by 30 cycles of 30 sec each at 95°C, 37°C and 1 min at 72°C with a final step of 5 min at 72°C. Genes were sequenced using the reverse primer and sequencing was performed by GATC-biotech (https://www.gatc-biotech.com). The partial *hsp60* gene sequences were aligned using Muscle (Edgar, 2004). Phylogenetic trees were built using PhyML applied to maximum-likelihood algorithm and GTR model as parameters (SPR, γ4, invariant site; Guindon et al., 2010). Reliability was assessed by the bootstrap method with 100 replicates. Circular tree figures were drawn using the online iTOL software package (Letunic and Bork, 2011).

### OsHV-1 DNA Quantification

The OsHV-1 DNA was quantified from ground tissues by real time PCR using the SYBR^®^Green chemistry (Labocea Quimper, France) as described previously (Pepin et al., 2008). The TaqMan^®^ chemistry, previously demonstrated to be more sensitive (Martenot et al., 2010) was used to quantify the herpes virus from hemolymph samples (Labéo, Laboratoire Frank Duncombe, Caen, France).

### Virulence Studies using Oysters

Bacteria were grown under constant agitation at 20°C for 24 h in Zobell media. One hundred mL of the pure culture (10^7^ cfu) was injected intramuscularly into anesthetized SPF oysters. The bacterial concentration was confirmed by conventional dilution plating on Zobell agar. After injection, the oysters were transferred to aquaria (20 oysters per aquarium of 2.5 L) containing 1 liter of aerated 5 μm-filtered seawater at 20°C, kept under static conditions for 24 h.

### Statistical Analysis

Statistical analysis was performed using the computing environment R Development Core Team. For all statistical comparisons, we first tested normality of distribution. For normal distributions we compared variables using Student’s *t*-test. For non-normal distributions, we compared variables using Wilcoxon rank test and tested correlations using the Spearman correlation coefficient.

## Results

### Rapid *Vibrio* Colonization followed by Viral Replication Precedes Oyster Death

**FIGURE 1:**
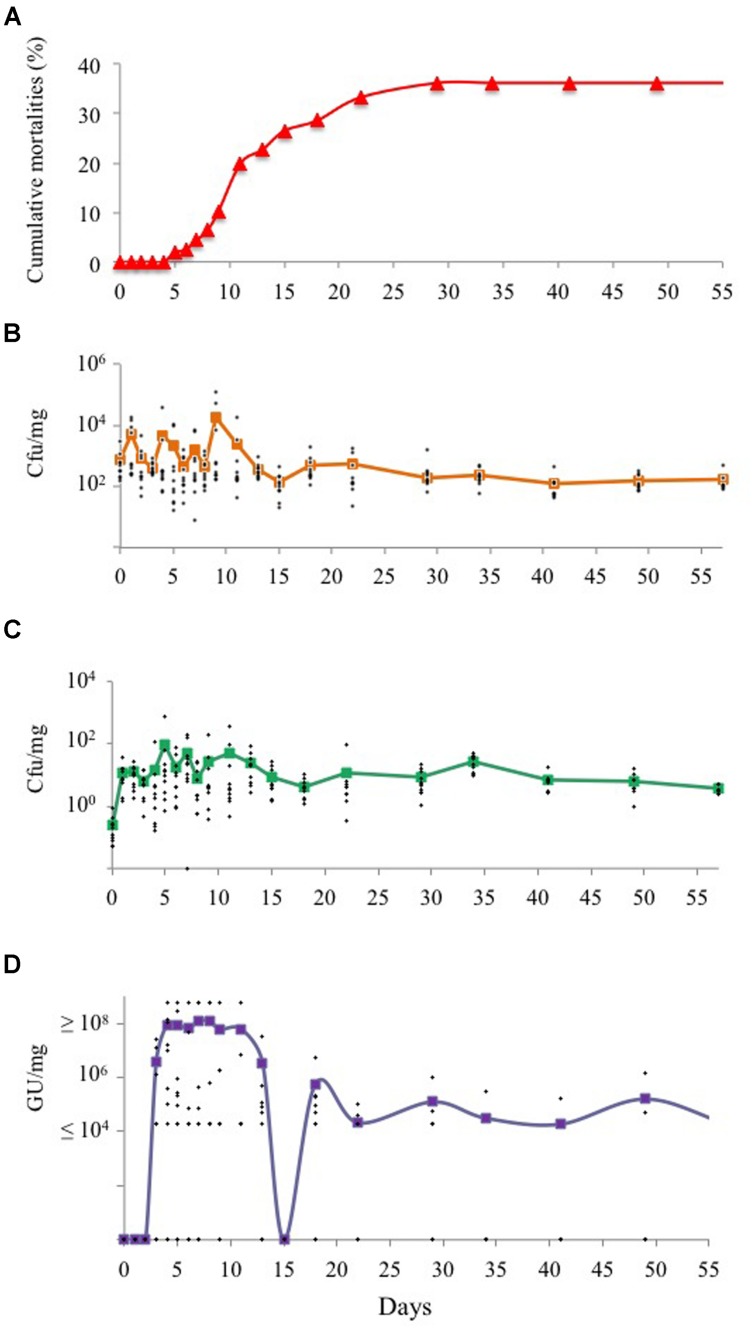
**Progression of multiple infections of oysters under field conditions.** Specific pathogen free (SPF) oysters were transferred to open seawater during a mortality outbreak. Cumulative mortalities were recorded daily (D0 to 57, x axis) and expressed in percentage (y axis, **A**). At different intervals, ten oysters were sacrificed, the total culturable bacterial **(B)** and *Vibrio* microbiota **(C)** were quantified (cfu/mg of tissues, y axis) by serial dilutions on Marine agar and TCBS, respectively. DNA was extracted from ground tissues and qPCR was performed to quantify the OsHV-1 load, expressed in Genome Unit per mg (GU/mg, y axis) of tissues **(D)**. In **(B–D)**, the lines indicate the average of 10 animals and dots the individual value.

Specific-pathogen-free oysters were maintained in the field during a disease outbreak. Mortalities began at day 5, increased dramatically until day 15, and then stabilized, reaching a final percentage of 36% cumulative mortalities after day 30 (**Figure 1A**). The starting concentration of cultivable bacterial microbiota was about 10^3^ cfu/mg of oyster-wet tissue prior to deployment in the field (**Figure 1B**) and did not change significantly after field exposure. However, the vibrio concentration did increase significantly after the first day in the field (*p* = 0.0001 using Wilcoxon test) and reached a maximum of 10^2^ cfu/mg (**Figure 1C**). After day 15, the vibrio concentration was stabilized to 10 cfu/mg. The ratio of vibrio to total cultivable microbiota was 10^-4^ in SPF oysters and 10^-2^ in animals maintained under field conditions. The OsHV-1 DNA was detected after day 3 and the amount of virus DNA reached a maximum of 10^8^ genome units (GUs)/mg between days 5–13 (**Figure 1D**). Surprisingly, at day 15, no viral DNA was detected from the 10 sampled oysters. Finally, from day 20 to the end of the experiment, the viral load was maintained around 10^5^ GU/mg. The inter-individual variability in microbial counts was higher during the first 15 days of exposure (**Figures 1B–D**). No correlation was found between the vibrio concentration and viral load. This result was confirmed by a Spearman’s test (*p*-value ≥ 0.05; R^2^ = 0.009). Altogether our results show that while diseased oysters are co-infected by vibrios and virus, the presence of the herpes virus does not seem to influence the infection by vibrios and vice versa.

### Oyster Mortalities Correlate with a Higher Quantity of Herpes DNA

**FIGURE 2:**
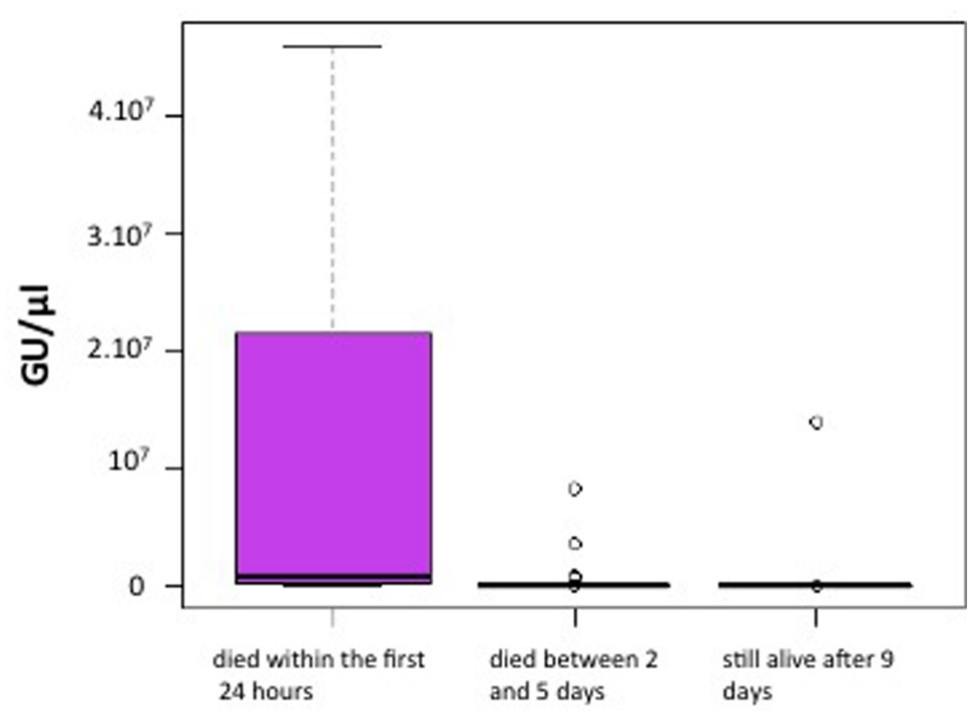
**OsHV-1 DNA load in naturally infected oysters brought back to the laboratory that died within the first 24 h or were still alive after 9 days.** SPF oysters were exposed to natural seawater in the field for 8 days and then returned to the laboratory. The hemolymph was non-destructively collected from marked individuals, then the oysters were kept in the laboratory to identify animals that died or survived. The OsHV-1 load is expressed in genome unit (GU)/μl of hemolymph (y axis).

We asked whether the quantity of OsHV-1 and vibrios could predict oyster mortalities. In two separate experiments in July 2014, SPF oysters were exposed to natural seawater for 8 days in the field and then returned to the laboratory before the onset of death. The hemolymph was non-destructively collected from marked individuals, and the oysters were kept in individual tanks in the laboratory to observe the onset of the disease. Mortalities started at day 1, reached 30% (first experiment) or 50% (second experiment) at day 5 and then leveled off (Supplementary Figure S1). The hemolymph sampling did not cause any effect on the mortality rate and disease kinetics as the sampled infected animals showed the same rate of mortality as the unsampled infected animals (Supplementary Figure S1). The quantity of OsHV-1 DNA detected in the hemolymph of animals that died within the first 24 h (**Figure 2**) was found to be significantly higher (up to 4 log units) than in the hemolymph of animals that were still alive after 9 days (pairwise comparison using Wilcoxon rank sum test; *p* = 0.0008). In contrast, the concentration of vibrios isolated from the hemolymph varied between individuals, but was not correlated with the mortality occurrence (data not shown).

Previous results have suggested that only a subset of vibrio strains are virulent by experimental infection (Lemire et al., 2014). Here, we investigated the diversity and pathogenicity of vibrios isolated from oysters that died and compared it to the diversity of those that survived. A total of 247 vibrio isolates sampled from the hemolymph of animals that died within the first 24 h and from animals that were still alive after 9 days were characterized by partial sequencing of a protein-coding gene (*hsp60).* Phylogenetic analysis allowed the grouping of 148/247 isolates into 11 clades (designated from A to K) with a bootstrap value >70% (Supplementary Figure S2). Some of these clades could be matched with named species using type strains: *V. ichthyoenteri* (A), *V. harveyi* (B), *V. mediterranei* (C), *V. fortis* (D), *V. chagasii* (E), *V. cyclitrophicus* (H), *V. tasmaniensis* (J), *V. breoganii* (K), and *V. crassostreae* (I). We did not observe a genotype signature in oysters that died with the first 24 h.

**FIGURE 3:**
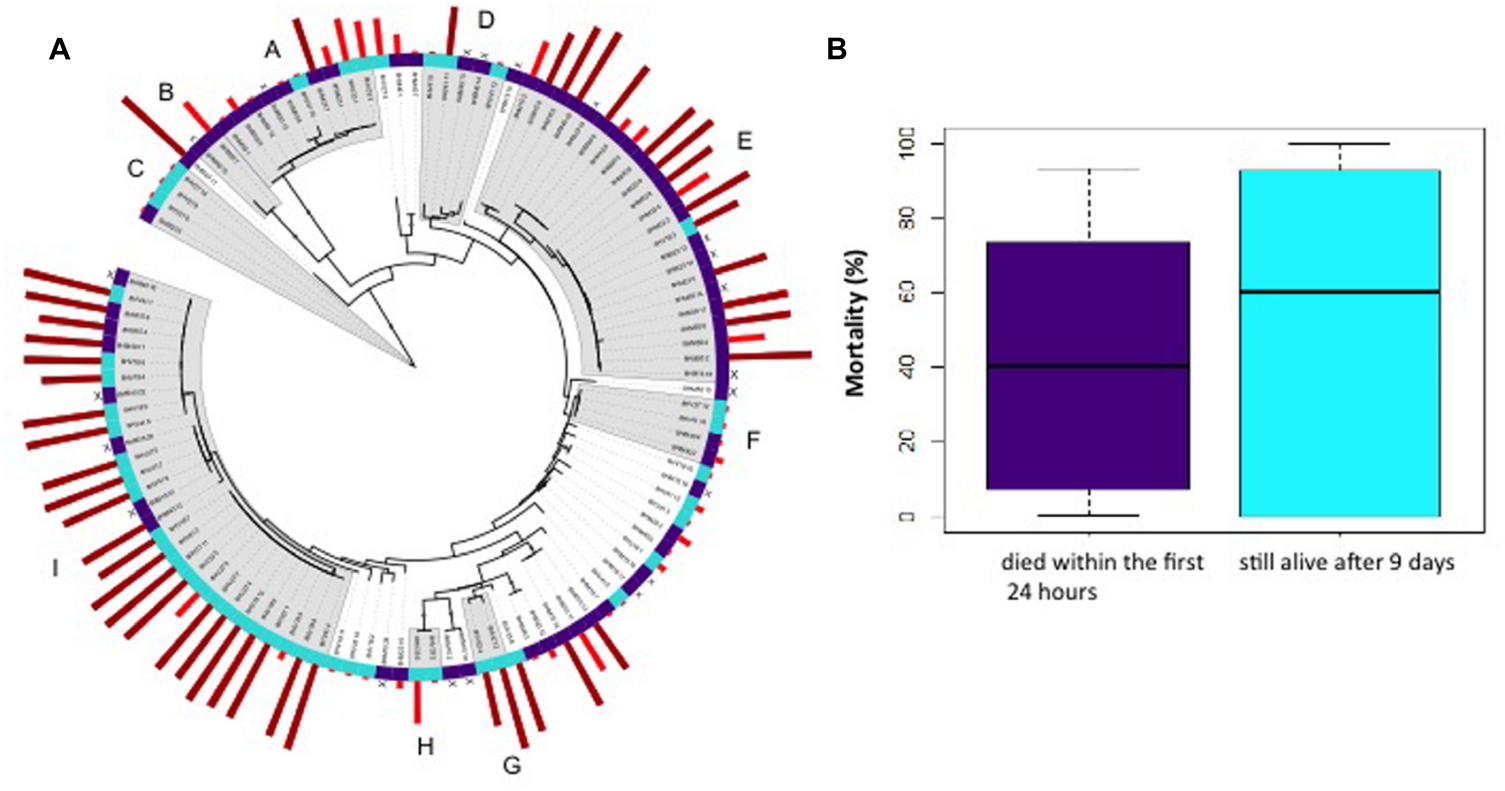
**Population structure and pathogenicity of vibrios isolated from naturally infected oysters.** The hemolymph was collected from four naturally infected oysters that were still alive after 9 days (turquoise) or four animals that died within the first 24 h (violet) in the laboratory and vibrios were isolated on selective media. **(A)** Virulence superimposed on the phylogeny of vibrio isolates inferred by maximum likelihood analysis of partial *hsp60* gene sequences (experiment 2, 29 July 2014). The outer ring indicates the % of mortalities obtained 24 h after oyster injection (brown bars > 50%; red bars < 50%). The inner ring indicates the origin of the strain, i.e., hemolymph of animal that survived after 9 days (turquoise) *versus* will die after 24 h (violet). Clades A to I had a bootstrap value > 70% and contain the type strains of *V. ichthyoenteri* (A), *V. harveyi* (B), *V. mediterranei* (C), *V. fortis* (D), *V. chagasii* (E), *V. cyclitrophicus* (H), and *V. crassostreae* (I). **(B)** Boxplot representation of oyster mortality rates induced by strains isolated from oysters that will survive after 9 days (turquoise) or will die after 24 h (violet). Mortalities were recorded at 24 h and expressed in percentage (y-axis). The percentages of mortalities were not found to be significantly different between the two conditions (Student test, *p* = 0.2513).

To address the pathogenic potential of individual strains, we used an injection model of infection, which allows for rapid infection in the laboratory (**Figure 3**). A total of 91 *Vibrio* strains, isolated in the second experiment (29 July), were injected individually into oysters at a concentration of 10^7^ cfu/animal (**Figure 3A**). The percentages of mortalities induced by strains isolated from the hemolymph of animals that died within the first 24 h and strains isolated from hemolymph of oysters that were still alive after 9 days were not found to be significantly different (Student test, *p* = 0.2513; **Figure 3B**). Half of the strains with >50% mortality rates belonged to the *hsp60* clade I (**Figure 3A**) and matched to the phylogenetically coherent virulent population described recently as *V. crassostreae* (Lemire et al., 2014). Hence, our results show that viral load is sufficient to predict mortalities.

### In the Absence of Bacteria, a High Load of Herpes Virus is not Sufficient for a Full Expression of the Disease

**FIGURE 4:**
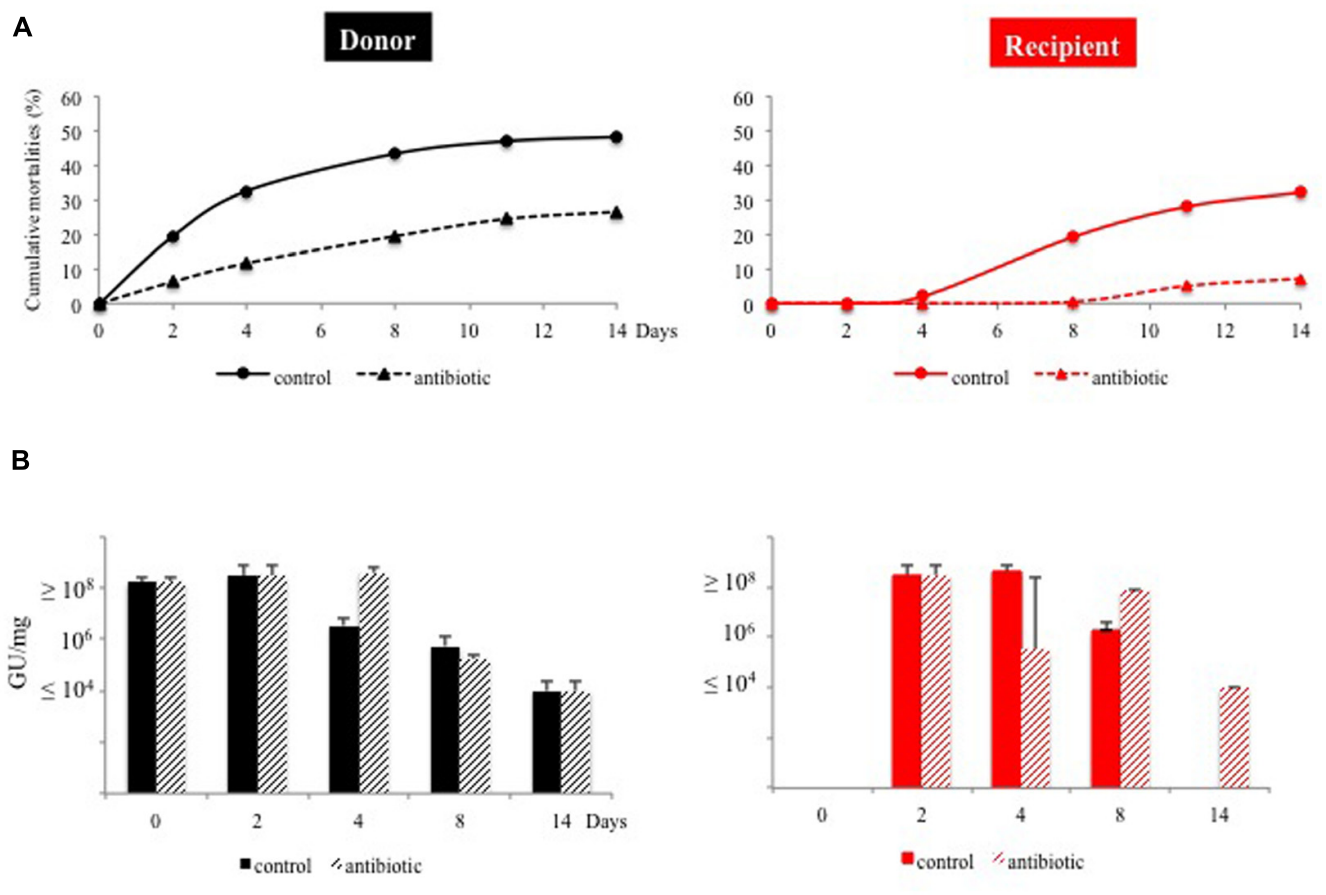
**Antibiotic treatment effect on disease expression and transmission.** SPF oysters were exposed to natural seawater under field conditions and then returned to the laboratory. These animals (donor, in black) were maintained in cohabitation with SPF oysters (recipient, in red) in the presence (hatched) or not (plain) of chloramphenicol for 14 days. **(A)** Mortalities were recorded each other day and expressed in percentage (left y-axis). **(B)** The OsHV-1 load, expressed in genome unit (GU) per mg of tissues was determined from five pooled animals.

Antibiotic treatments are typically used to investigate the role of bacteria in a specific disease. SPF oysters were exposed to natural seawater in the field and then returned to the laboratory after 10 days (see Material and Methods). These animals (referred to as “donors”) were kept together with SPF oysters (referred to as “recipients”) in the presence or absence of chloramphenicol for 14 days. In the absence of the antibiotic, donor mortalities started at day 2 and reached a cumulative value of 50%. In comparison, recipients began to die at day 8 and reached a cumulative mortality of 36% (**Figure 4A**). The antibiotic treatment resulted in a 2- and 4-fold decrease in mortality for donors and recipients, respectively, demonstrating a role of bacteria in the disease transmission and development. While chloramphenicol exposure completely removed the cultivable vibrio microbiota in the oyster tissues (data not shown), no significant effect was observed on the OsHV-1 DNA quantity detected in the donors or the recipients until day 8 (**Figure 4B**).

### Oysters are Reservoirs of Pathogens

The results above demonstrate that oysters can asymptomatically host putative pathogens, raising concern about the role of oysters as pathogen reservoirs. Using a cohabitation experiment, we asked whether oysters carry putative pathogens at temperatures of <16°C (i.e., excluding mortality context). French oysters are cultivated to the size of “spats,” the point at which they attach themselves to a substrate. These spats originated from natural settlement zones (wild seeds) or were produced in hatchery (hatched seeds). Wild adult oysters (i.e., not farmed) can also be collected from the environment. Hence, we also determined whether the geographic origin, age, and farming of oysters influence the disease(s) transmission.

The first experiment was performed with juvenile oysters originated from wild seed collected in 6 different geographic areas as donors, and 3-months-old SPF hatch oysters as recipients (**Table 1**). Over the course of the experiment (30 days), the hatched animals did not die on their own (not shown). On the contrary, death occurred in oysters originated from wild seeds collected in four regions (Arcachon, Pertuis Charentais, Bay of Vilaine, and Bay of Brest). In this case, 11/15 batches of juveniles originating from these regions were able to transmit the disease to the recipients. Finally, six batches originating from two regions (Thau and Bay of Bourgneuf) did not show significant mortality rates and did not cause mortality in the recipients. Mortalities always coincided with the detection of OsHV-1 in both donors and recipients. Interestingly, in 7/21 batches of donors, the virus was not detected at the beginning of the experiment but was found at the end of the trial, confirming that a thermal stress in the mesocosm (see Materials and Methods) can be used to highlight viral infection (Petton et al., 2015).

**Table 1:** Cohabitation experiments performed for 30 days with juvenile oysters collected in six different geographic areas as donors and 3 months-old-SPF oysters as recipients (nd, not detected; d, detected).

Donors					Recipients		
Geographic origin	Wild seed collecting sector	Mortality (%) at day 30	OsHV-1 μvar at day 0	OsHV-1 μvar at day 30	Mortality (%) at day 30	OsHV-1 μvar at day 0	OsHV-1 μvar at day 30
Thau	Marseillan	3 ± 1	nd	d	0	nd	nd
	Loupian	6	nd	nd	0	nd	nd
	Loupian	2 ± 1	nd	nd	0	nd	nd
Arcachon	Lucarnan	25 ± 1	nd	d	46 ± 1	nd	d
	Villa Algèrienne	22 ± 25	nd	d	13 ± 17	nd	d
	Graouères	5 ± 5	nd	nd	0	nd	nd
	Comprian	19 ± 0	nd	d	0	nd	d
	Bélisaire	16 ± 2	d	d	35 ± 9	nd	d
Pertuis Charentais	Les Longès	1 ± 0	nd	nd	0	nd	nd
	Seudre	67 ± 4	d	d	42 ± 5	nd	d
	Mouclières	0	nd	nd	0	nd	nd
Bay of Bourgneuf	Les Moutiers	0	d	d	0	nd	nd
	Bernerie Nord	0	d	d	0	nd	d
	Bernene Sud	0	nd	d	0	nd	nd
Bay of Vilaine	Tréguier (gros)	54 ± 1	nd	d	63 ± 5	nd	d
	Tréguier (petit)	27 ± 17	nd	d	16 ± 22	nd	d
Bay of Brest	Le Pryoldi	56 ± 1	d	d	36 ± 10	nd	d
	Mengleuz	57 ± 4	d	d	39 ± 6	nd	d
	Lomergat	68 ± 1	d	d	39 ± 9	nd	d
	Pte du Château	79 ± 6	d	d	49 ± 3	nd	d
	Pte du Château	80 ± 2	d	d	51 ± 3	nd	d

The second experiment was performed with farmed or wild adults (>18 months) sampled from four different geographic areas as donors, and 3-, 6-, and 15-months-old SPF oysters as recipients (**Table 2**). Only 1/10 sets of wild oysters expressed mortalities and transmitted the disease to the recipients. On the contrary, 13/18 (72%) batches of farmed oysters originating from the four regions showed mortalities and caused the disease in the recipients. A Wilcoxon test confirmed that the farmed animals had a significantly higher death rate and transmitted more of the disease than the wild oysters (*p* = 0.0007). In all of these cases, the mortality rate was negatively correlated with the age of recipient animals. Interestingly, OsHV-1 was detected only once in the recipients (not shown), suggesting no significant role for the virus in the disease transmetted by adults (**Table 2**). Altogether, our results demonstrate that oysters are a reservoir of putative pathogens. Only juvenile oysters were shown to carry and transmit a detectable amount of OsHV-1, while adult animals carried infectious agents that are able to induce mortality of juveniles in the absence of the virus.

**Table 2:** Cohabitation experiments performed for 30 days with farmed or wild adults sampled in 4 different geographic areas as donors and 3-, 6-, and 15-months-old SPF oysters as recipients.

Donors				Recipients		
Geographic origin	Collecting sector	Farmed/wild	Mortality (%)	Mortality (%) 3 months	Mortality (%) 6 months	Mortality (%) 15 months
Bay of Veys	Géfosse	Farmed	38	64	43	29
Aber Benoit	Beg ar Vill	Farmed	20	64	26	20
	Beg ar Vill	Farmed	31	57	30	24
	Beg ar Vill	Farmed	59	55	33	28
	Beg ar Vill	Farmed	46	48	26	21
	Beg ar Vill	Farmed	20	63	37	28
Bay of Bourgneuf	La Coupelasse	Farmed	5		5	8
	Cob	Farmed	64		51	17
	Roche aux Bretons	Farmed	69		25	25
	Grill Nod	Farmed	54		28	29
	Graisseloup	Farmed	57		33	25
	La Coupelasse	Wild	2		0	0
	Cob	Wild	0		1	0
	Graisseloup	Wild	1		0	0
	Roche aux Bretons	Wild	2		0	0
Bay of Brest	Pte du Château	Farmed	0	0	0	0
	Pte du Château	Farmed	8	28	7	17
	Pte du Château	Farmed	0	0	0	0
	Pte du Château	Farmed	5	0	0	0
	Pte du Château	Farmed	0	0	0	1
	Pte du Château	Farmed	17	66	23	20
	Pte du Château	Farmed	91	78	59	54
	Pte du Château	Farmed	3	70	14	22
	Le Prioldy	Wild	3	0	0	0
	Le Loc’h	Wild	0	0	0	0
	Pte du Château	Wild	31	45	19	15
	Pte du Château	Wild	0	0	0	0
	Pte du Château	Wild	0	0	0	0
	Pte du Château	Wild	8	57	7	0
	Pte du Château	Wild	1	1	0	0

## Discussion

In this study, we investigated the respective roles of herpes virus and vibrios in the juvenile oyster disease by exposing SPF oysters in the field during a disease outbreak. Prior to field exposure, the cultivable fraction of the SPF oyster microbiota was determined to be 10^3^ cfu/mg tissue, and a low vibrio presence (≤1 cfu/mg) was detected in these animals (**Figure 1**). Hence, these animals represent a suitable model to investigate the role of each pathogenic agent in the disease process, both independently and together.

The limited sensitivity in detecting viral DNA (10^4^ GU/mg tissue), prevented the monitoring of initial infection kinetics for the animals. Hence, the detection of viral DNA after 3 days of exposition under field conditions likely resulted from the viral replication culminating at 10^8^ GU/mg (**Figure 1**). While the mortalities stabilized after 15 days, the viral load was maintained at 10^4^ GU/mg, and can thus be considered as an asymptomatic carriage. Whether this load corresponds to a latent phase of the virus or to a limited shedding of particles remains to be determined, but our results highlight the interest of our model to explore the herpes virus biological cycle (i.e., latent and lytic cycle; cellular tropism). Whereas previous data correlating the virus load with mortalities have been based on moribund animal sampling (Sauvage et al., 2009; Oden et al., 2011), we performed a non-destructive collection of hemolymph and showed that viral DNA quantification (>10^7^ GU/mg) can predict the mortalities of juvenile oysters (**Figure 2**). However, when using an antibiotic treatment, we also demonstrated that in the absence of bacteria, a load of herpes virus ≥10^8^ GU/mg is not sufficient to induce oyster mortalities in a range classically ascribed to this disease (**Figure 4**). These results suggest that viral replication may be a consequence rather than the cause of the disease. Recently, Green and Montagnani (Green et al., 2014) observed that a poly I:C treatment decreases the OsHV-1 replication in *C. gigas* infected by injection. Hence developing poly I:C and/or RNA interference approaches (Labreuche and Warr, 2013; Huvet et al., 2015) in oysters would be of prime interest to decipher the direct role of this virus in the disease process.

Having shown that oysters are naturally co-infected by the herpes virus and vibrios, we next ask if a synergy between these microbes is fundamental to the disease. In a first scenario, oysters infected with herpes may be more susceptible to subsequent *Vibrio* infection. However, this hypothesis is contradicted by the fact that we did not observe a correlation between the herpes load and vibrio concentration at the individual level. In a second scenario, oysters infected with *Vibrio* may be more susceptible to herpes replication. However, as in antibiotic treated oysters the viral load is similar to that of untreated animals (**Figure 4**), this scenario seems also to be unlikely. In any case, diseased oysters that are co-infected by several putative pathogens (virus and bacteria) should sustain much greater losses.

Since we aimed at differentiating the roles of *Vibrio* spp. and the herpes virus as causative agents of oyster deaths, we used an antibiotic treatment and highlighted an essential role of bacteria in the disease process (**Figure 4**). As chloramphenicol treatment is not restricted to vibrio, the next step will be to demonstrate that vibrio(s) is (are) the causative agent(s) of the death. We observed that the quantity, genotype and virulence of vibrios (determined by the injection of single strain) cannot predict the mortalities (**Figure 3**). Our results seem to contradict a recent study that describes the microbial communities in oysters challenged by a virulent vibrio strain (Lokmer and Wegner, 2015). Lokmer and Wegner found that dead and moribund oysters displayed signs of community structure disruption characterized by a very low diversity and proliferation of few taxons. However, their analysis used barcoded 16S rRNA 454 amplicon sequencing, the genetic resolution of which is not fine scale enough to analyze the vibrio diversity. Furthermore, the bacterial challenge was based on single strain injection, which does not capture the complexity of infection within the natural environment as described in the present study. We previously hypothesized that *Vibrio* diversity may have significance in the disease onset. In a first study we showed that heightened virulence is observed when combination of virulent strains are used in experimental infections, suggesting that virulence of vibrio results from a combination of factors, each associated with different strains within the population (Gay et al., 2004). More recently, we demonstrated that naturally infected oysters initially contain a large proportion of non-virulent strains, and that these are progressively replaced by a virulent population that comprise ∼50% of the bacterial isolates at the peak of mortality (Lemire et al., 2014). We suspected that this result reflects a contribution of the non-virulent strains to the development of disease. We further demonstrated that the presence of non-virulent bacteria dramatically increases the virulence of a virulent strain at low doses. Thus, non-virulent strains must have some features that contribute either directly or indirectly to the pathogenicity of virulent isolates. In the future we will investigate the significance of *Vibrio* population assembly in diseased oysters with emphasis on cooperative behavior and weapons sharing that may be an important feature of the disease.

From the data collected in this study, the oyster physiology seems to be a key feature of this disease. We observed that when oysters are naturally co-infected by the herpes virus and pathogenic vibrios, only 30% to 50% of animals died. This suggests that viral replication and/or the vibrio’s pathogenic effect depends on oyster susceptibility. This susceptibility may result from environmental factors (acquired susceptibility) and/or genetic traits (innate susceptibility). It may implicate the physiology of the animal itself and its associated microbial communities (Lokmer and Wegner, 2015). It has been demonstrated that resistance toward oyster juvenile disease has a genetic basis (Huvet et al., 2004; Segarra et al., 2014). More recently, Wendling and Wegner suggested that a simple genetic resistance mechanism of the oyster is matched to a common virulence mechanism shared by sympatric *Vibrio* strains (Wendling and Wegner, 2015). In the future, we will investigate the genetic bases of oyster susceptibility to the vibrio isolated in the present study.

Both adult and juvenile oysters were shown to act as reservoirs of pathogens which were able to induce mortalities of young animals. Only the juveniles were demonstrated to transmit the herpes virus. This suggests that the diseases transmitted by the juveniles or by the adults are distinct. However, it also demonstrates that the virus infection is not a prerequisite for juvenile mortality outbreaks. Farmed animals seem to present more risk of disease transmission than wild animals. In addition, the geographic origin of oysters influences the carriage of pathogen. Though our data suggest that some farming zones are less infected than others they also indicate that movements of animals between ecosystems are very noxious in terms of disease-spreading. Beyond the environmental factors that are known to potentially influence mechanisms of disease transmission in marine systems, i.e., hydrodynamics, biomass of infected animals (Petton et al., 2015), identifying other potential reservoirs of pathogens and the source(s) of the disease is a future avenue to qualify infected vs. non-infected areas.

## Conflict of Interest Statement

The authors declare that the research was conducted in the absence of any commercial or financial relationships that could be construed as a potential conflict of interest.
