# Supplementary figures and images for: Crassostrea gigas mortality in France: the usual suspect, a herpes virus, may not be the killer in this polymicrobial opportunistic disease
Source: Front Microbiol. 2015 Jul 6;6:686. doi: 10.3389/fmicb.2015.00686 (PMC4491618; doi:10.3389/fmicb.2015.00686)

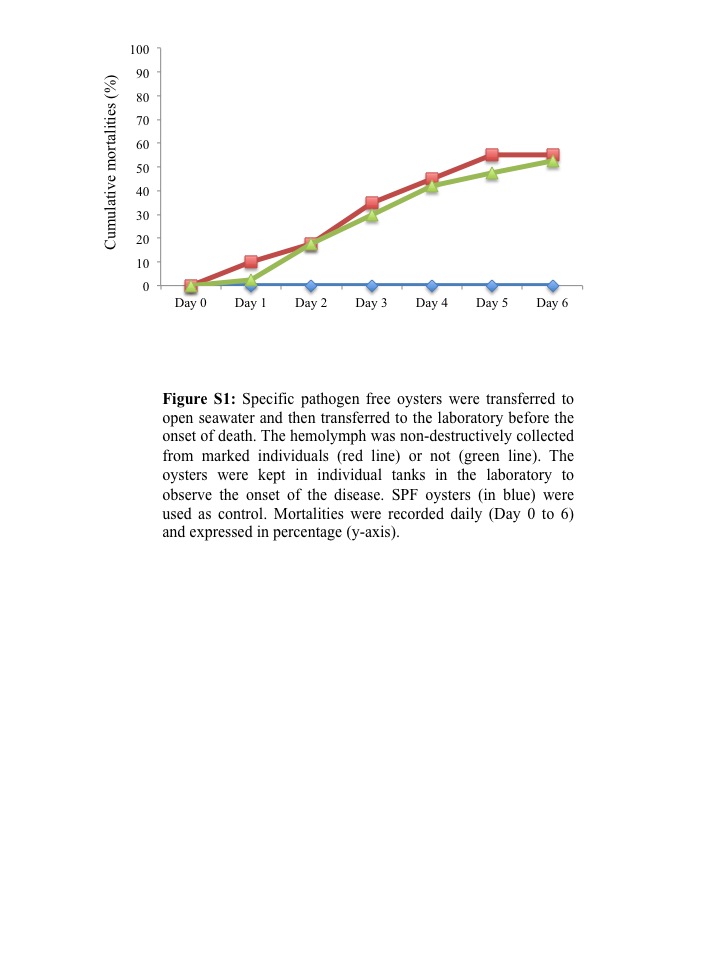

Supplement: Supplementary file 1 [file Image_1.JPEG]

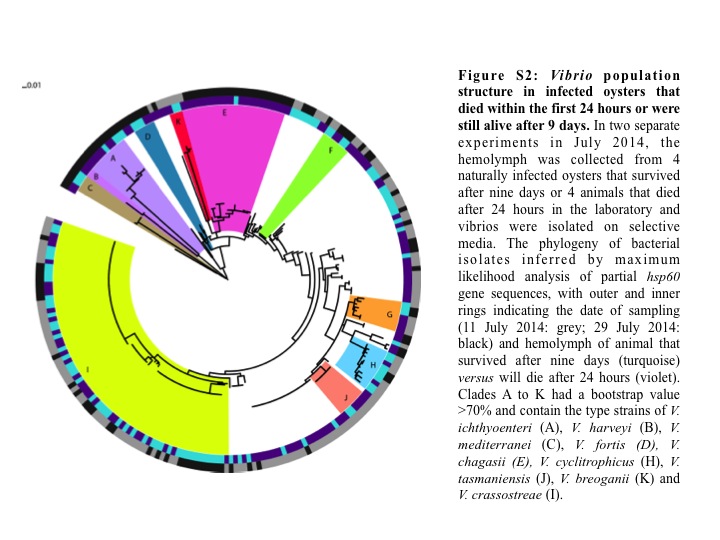

Supplement: Supplementary file 2 [file Image_2.JPEG]
